# Unlocking the Secrets of *Streptococcus suis*: A peptidomics comparison of virulent and non-virulent serotypes 2, 14, 18, and 19

**DOI:** 10.1371/journal.pone.0287639

**Published:** 2023-06-29

**Authors:** Chadaporn Chaiden, Janthima Jaresitthikunchai, Narumon Phaonakrop, Sittiruk Roytrakul, Anusak Kerdsin, Suphachai Nuanualsuwan

**Affiliations:** 1 Faculty of Veterinary Sciences, Department of Veterinary Public Health, Chulalongkorn University, Bangkok, Thailand; 2 Faculty of Veterinary Science, Department of Veterinary Public Health, Center of Excellence for Food and Water Risk Analysis (FAWRA), Chulalongkorn University, Bangkok, Thailand; 3 Functional Proteomics Technology Laboratory, Functional Ingredients and Food Innovation Research Group, National Center for Genetic Engineering and Biotechnology, National Science and Technology for Development Agency, Pathum Thani, Thailand; 4 Faculty of Public Health, Kasetsart University Chalermphrakiat Sakon Nakhon Province Campus, Sakon Nakhon, Thailand; BOKU: Universitat fur Bodenkultur Wien, AUSTRIA

## Abstract

*Streptococcus suis* (*S*. *suis*) is an important bacterial pathogen, that causes serious infections in humans and pigs. Although numerous virulence factors have been proposed, their particular role in pathogenesis is still inconclusive. The current study explored putative peptides responsible for the virulence of *S*. *suis* serotype 2 (SS2). Thus, the peptidome of highly virulent SS2, less prevalent SS14, and rarely reported serotypes SS18 and SS19 were comparatively analyzed using a high-performance liquid chromatography-mass spectrometry method (LC-MS/MS). Six serotype-specific peptides, 2,3,4,5-tetrahydropyridine-2,6-dicarboxylate N-acetyltransferase (DapH), alanine racemase (Alr), CCA-adding enzyme (CCA), peptide chain release factor 3 (RF3), ATP synthase subunit delta (F0F1-ATPases) and aspartate carbamoyltransferase (ATCase), were expressed moderately to highly only in the SS2 peptidome with *p*-values of less than 0.05. Some of these proteins are responsible for bacterial cellular stability; especially, Alr was highly expressed in the SS2 peptidome and is associated with peptidoglycan biosynthesis and bacterial cell wall formation. This study indicated that these serotype-specific peptides, which were significantly expressed by virulent SS2, could serve as putative virulence factors to promote its competitiveness with other coexistences in a particular condition. Further *in vivo* studies of these peptides should be performed to confirm the virulence roles of these identified peptides.

## Introduction

*Streptococcus suis* (*S*. *suis*) is an important bacterial pathogen, that causes arthritis, meningitis, pneumonia, septicemia, endocarditis, and polyserositis in the pig. In addition, it is a zoonotic pathogen responsible for severe streptococcal infection in humans especially people with a history of exposure to diseased pigs or consumption of contaminated pork. Meningitis, sepsis, arthritis, endocarditis, and endophthalmitis are the main clinical syndromes with consequences of loss of hearing and vestibular dysfunction [1]. Formerly, *S*. *suis* was divided by its capsular polysaccharide into 35 serotypes [2] and then was later re-classified into 29 serotypes [3–5]. *S*. *suis* serotype 2 (SS2) is the most prevalent in humans and pigs worldwide, while some other *S*. *suis* serotypes such as SS14 have much less frequently caused human infections [6]. More than 1,600 cases of streptococcal infection were reported worldwide including in Europe, Asia, North America, South America, Australia, and New Zealand [6–9]. Since the large outbreak in China in 2005, Thailand and Vietnam appear to be endemic areas for this pathogen [10,11]. Regarding virulence factors, it is challenging to address the particular mechanism since *S*. *suis* strains possess very high-diversity [12]. Over 100 putative virulence factors have been described and some have been widely studied and are known for *S*. *suis* pathogenesis, such as capsular polysaccharide (CPS), suilysin (SLY), enolase (Eno), muramidase-released protein (MRP), factor H binding protein (Fhb), and extracellular protein factor (EF) [13–17]. The particular roles of these recognized and unrevealed virulence factors in the host pathogenesis would be useful to know. Hence, many researchers have managed to investigate these unknown factors. Over recent decades, proteomic techniques have been used to explore the microbial proteins and peptides expressed by bacterial pathogens, such as *Salmonella enterica* and *Staphylococcus aureus*. As for the techniques capable of identifying the putative virulence factors, the proteome of *S*. *suis* has also been studied to consolidate their particular roles of putative virulence factors in the host as well [18–21]. Various putative virulence factors of SS2 have been proposed such as, acetaldehyde-CoA/alcohol dehydrogenase (adhE), catabolite control protein A (Ccp A), leucyl aminopeptidase (LAP), enolase (Eno), and endopeptidase [19,22–24]. According to our previous work, peptidomics analysis revealed the role of the ABC-type phosphate transport system (SSU05_1106) and 30S ribosomal protein S2 (rpsB) in the survival of *S*. *suis* in a growth medium supplemented with 5% sheep blood [18], since some bacteria or yeast are adaptive or responsive to different environments or growth conditions [25–28].

In the present study, peptides of highly virulent SS2, less prevalent SS14, and rarely reported serotypes SS18 and SS19 were cultured in a growth medium without blood supplement and comparatively analyzed by a high-performance liquid chromatography-mass spectrometry (LC-MS/MS) method. The modified extraction method for *S*. *suis* serotyping with matrix-assisted laser desorption/ionization time-of-flight mass spectrometry (MALDI-TOF-MS) was chosen since this method provides high quality and quantity of the extracted peptides [29]. The objective of this study was to explore the putative peptides in the peptidomes of SS2, SS14, SS18, and SS19 that are possibly involved in the virulence of *S*. *suis*, especially highly virulent SS2.

## Materials and methods

### Bacterial strains

The highly virulent *S*. *suis* serotype 2 (SS2) and the less prevalent *S*. *suis* serotype 14 (SS14), originally isolated from some diseased pigs were ATCC 700794 and 13730, respectively. The rarely reported *S*. *suis* serotypes, isolated from some healthy pigs, were *S*. *suis* serotype 18 (SS18) NT77 and *S*. *suis* serotype 19 (SS19) 42A, respectively. These four references *S*. *suis* serotypes were cultured on Columbia blood agar (Difco Laboratories, Detroit, MI, USA) with 5% (v/v) sheep’s blood at 37°C for 24 h. A GasPak Anaerobic System (Mitsubishi Gas Chemical Co., Inc., Tokyo, Japan) was used to generate the anaerobic condition. The 16S rRNA gene was sequenced to confirm the *S*. *suis* serotype. The primers used for sequencing were F1 and R13 primers [30], the accession numbers of SS2, SS14, SS18, and SS19 are LS483418.1, AF009489.1, AF009493.1, and AF009494.1, respectively. Then, the bacterial colonies were cultured in a Todd–Hewitt broth (THB) (Difco Laboratories, Detroit, MI, USA) and preserved with 20% glycerol at −80°C for further study.

### Preparation of peptidome

*S*. *suis* serotypes SS2, SS14, SS18, and SS19 were individually cultured on Columbia blood agar (Difco Laboratories, Detroit, MI, USA) with 5% (v/v) sheep’s blood at 37°C in the anaerobic condition for 24 h. The bacterial peptides were extracted according to a previous study [29]. Briefly, all colonies of *S*. *suis* grown on the blood agar plate were collected. The bacterial peptides were then denatured with 70% (v/v) ethanol and centrifuged at 11,000 g for 5 min and then the supernatant was discarded. A mixture of 5% (v/v) trifluoroacetic acid (TFA) with absolute acetonitrile (ACN) was added. The suspension was dissolved before centrifugation at 150 g for 30 min. The ACN was removed and the sample was resuspended with 0.1% (v/v) formic acid. A Lowry assay was used to determine the peptide concentration [31].

### LC-MS/ MS

An HCTUltra PTM Discovery System (Bruker Daltonics Ltd., Bremen, Germany) coupled with an UltiMate 3000 LC System (Dionex Ltd., Camberley, UK) was used to analyze peptides in the samples. The peptide samples were separated on a nanocolumn (PepSwift monolithic column 100 μm i.d. x 50 mm) using reversed-phase high-performance liquid chromatography (HPLC). Two eluents were used. Eluent A was 0.1% formic acid and eluent B was 50% ACN in water containing 0.1% formic acid. An eluent B gradient in the range of 4 to 70% was used to elute peptides at a constant flow rate of 1,000 nL/min for 7.5 min. A CaptiveSpray was used to generate electrospray ionization at 1.6 kV. Nitrogen as a collision gas was used with a flow rate of 50 L/h. The collision-induced-dissociation product ion mass spectra were obtained. Positive-ion mode at 2 Hz over the (m/z) range 150–2200 was used to collect Mass spectra (MS) and MS/MS. The collision energy was adjusted to 10 eV as a function of the m/z value. All samples were triplicated before being analyzed by LC-MS.

### Peptidomics data analysis

The hypothetical peptides derived from MS/MS were simultaneously analyzed and quantified using the DeCyder MS Differential Analysis Software (DeCyderMS, GE Healthcare). The protein identification was performed by matching the analyzed MS/MS signal data with the Uniprot database using the Mascot software (Matrix Science, London, UK). The search parameters were taxonomy (*Streptococcus suis*), enzyme (NoCleave), variable modifications (oxidation of methionine residues), mass values (monoisotopic), protein mass (unrestricted), peptide mass tolerance (1.2 Da), fragment mass tolerance (± 0.6 Da), peptide charge state (1+, 2+ and 3+), and missed cleavages (3). The full sequence of the main fragmentation series of the MS/MS spectra was determined using the Mascot software. The level of peptide expressions was illustrated by a Hierarchical clustering heat map using Multiple Experiment Viewer version 4.9.0, Mev [32]. The distributions of differentially expressed peptides among peptidomes of four reference *S*. *suis* serotypes were displayed using Venn Diagrams with jvenn [33]. The particular protein annotation was acquired using UniProtKB/Swiss-Prot entries (http://www.uniprot.org/ accessed on 29 July 2020). Furthermore, the identified proteins were classified by the PANTHER classification system version 17.0 (http://www.pantherdb.org/ accessed on 26 February 2023) [34]. STITCH 5.0 (http://stitch.embl.de/ accessed on December 10, 2022) was used to determine the relationships between identified proteins of four reference *S*. *suis* serotypes [35].

### Statistical analysis

The significantly different peptide peaks were evaluated using a Student’s *t*-test and one-way analysis of variance (ANOVA), incorporated into the DeCyder MS Differential Analysis Software. The statistical significance level was set at a *p*-value of less than 0.05. The Pearson correlation was used for Hierarchical clustering heat map.

## Results

### Identified peptides

**Fig 1:**
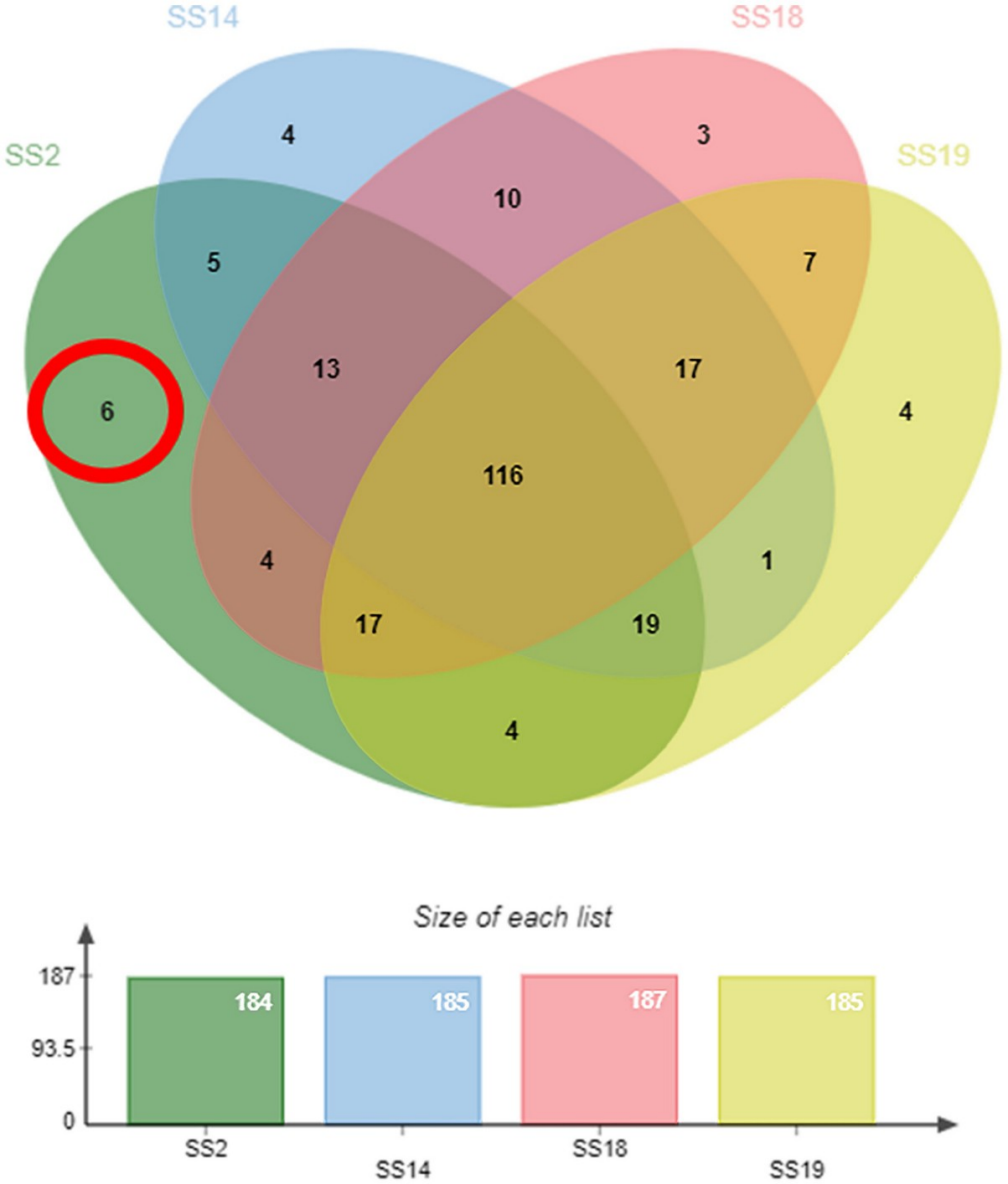
Distribution of peptides identified in peptidomes of SS2, SS14 SS18, and SS19.

**Fig 2:**
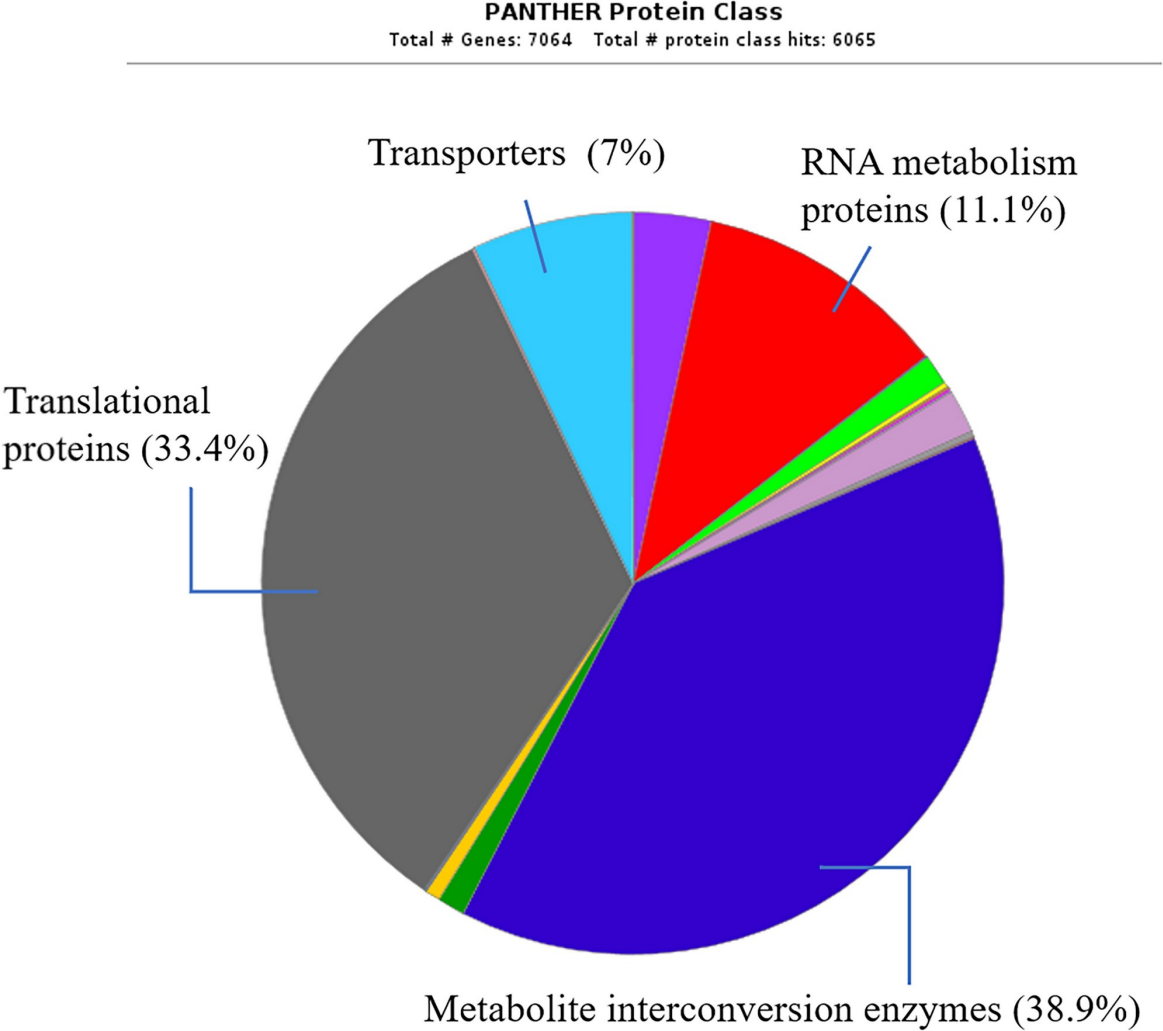
Proteins classes of the identified peptides in peptidomes of SS2, SS14, SS18, and SS19.

Various sizes of peptides (*n* = 229) in the range 360–3,700 Da were derived from the peptidomes of all four reference *S*. *suis* serotypes. A total of 187 peptides of SS18 were identified, followed by 185 peptides of SS14 and SS19, and 184 peptides belonging to SS2 as shown in the jvenn (Fig 1). Even though each *S*. *suis* serotype had almost 200 identified peptides, all four reference *S*. *suis* serotypes shared as many as 116 peptides in common. In terms of the protein class [34], the identified peptides mainly belong to the metabolite interconversion enzymes (38.9%), followed by the translational proteins (33.4%), RNA metabolism proteins (11.1%), and transporters (7%) (Fig 2). The complete list of peptides is provided in the supporting information (S1 Table). Four peptides, methionyl-tRNA formyltransferase (fMet), 4-hydroxy-tetrahydrodipicolinate reductase (HTPA reductase), hydroxyethylthiazole kinase (TH kinase), and ribonuclease Y (RNase Y), were solely identified in SS14. Three peptides only derived from SS18 were ribosome-binding factor A (RbfA), UvrABC system protein B (UvrB), GTPase Obg (GTP-binding protein Obg) while four peptides specifically derived from SS19 were serine/threonine transporter (SstT), deoxyribose-phosphate aldolase (DERA), SsrA-binding protein (Small protein B) and phosphoenolpyruvate carboxylase (PEPC) as shown in Table 1.

**Table 1:** Identified proteins derived from peptidomes of SS14, SS18, or SS19.

*S*. *suis*	Gene	Protein
SS14	*fmt*	methionyl-tRNA formyltransferase
	*dapB*	4-hydroxy-tetrahydrodipicolinate reductase
	*thi*M	hydroxyethylthiazole kinase
	*rny*	ribonuclease Y
SS18	*rbfA*	ribosome-binding factor A
	*uvrB*	UvrABC system protein B
	*obg*	GTP-binding protein Obg
SS19	*sstT*	serine/threonine transporter SstT
	*deoC*	deoxyribose-phosphate aldolase
	*smpB*	SsrA-binding protein
	*ppc*	phosphoenolpyruvate carboxylase

Virulent SS2 and SS14 shared five peptides in common while rarely reported serotypes SS18 and SS19 co-expressed the same seven peptides (Table 2). We noted that six unique peptides were found only in the peptidome of highly virulent SS2. These were 2,3,4,5-tetrahydropyridine-2,6-dicarboxylate N-acetyltransferase (DapH), alanine racemase (Alr), CCA-adding enzyme (CCA), peptide chain release factor 3 (RF3), ATP synthase subunit delta (F0F1-ATPases) and aspartate carbamoyltransferase (ATCase), as shown in Fig 1 and Table 3.

**Table 2:** Co-identified proteins derived from virulent (SS2 and SS14) and rarely reported (SS18 and SS19) peptidomes of *S*. *suis*.

*S*. *suis*	Gene	Protein
SS2 and SS14	*purM*	phosphoribosylformylglycinamidine cyclo-ligase
	*rpmH*	50S ribosomal protein L34
	*ruvA*	holliday junction ATP-dependent DNA helicase RuvA
	*mnmA*	tRNA-specific 2-thiouridylase MnmA
	*divIB*	cell division protein DivIB
SS18 and SS19	*dtd*	D-aminoacyl-tRNA deacylase
	*rplF*	50S ribosomal protein L6
	*atpC*	ATP synthase epsilon chain
	*SSU05_0620*	nucleotide-binding protein
	*glyA*	serine hydroxymethyltransferase
	*lacB*	galactose-6-phosphate isomerase subunit LacB
	*scpA*	segregation and condensation protein A

**Table 3:** Identified proteins derived from peptides only expressed in the peptidome of highly virulent SS2.

Gene	Protein (Amino-Acid Length)	Function
*dapH*	2,3,4,5-tetrahydropyridine-2,6-dicarboxylate N-acetyltransferase (232)	lysine biosynthesis
*alr*	alanine racemase (367)	D-alanine biosynthesis
*cca*	the CCA-adding enzyme (403)	tRNA binding
*prfC*	peptide chain release factor 3 (514)	protein biosynthesis
*atpH*	ATP synthase subunit delta (177)	proton-transporting ATP synthase activity
*pyrB*	aspartate carbamoyltransferase (307)	catalytic Activity

### Peptide expression

**Fig 3:**
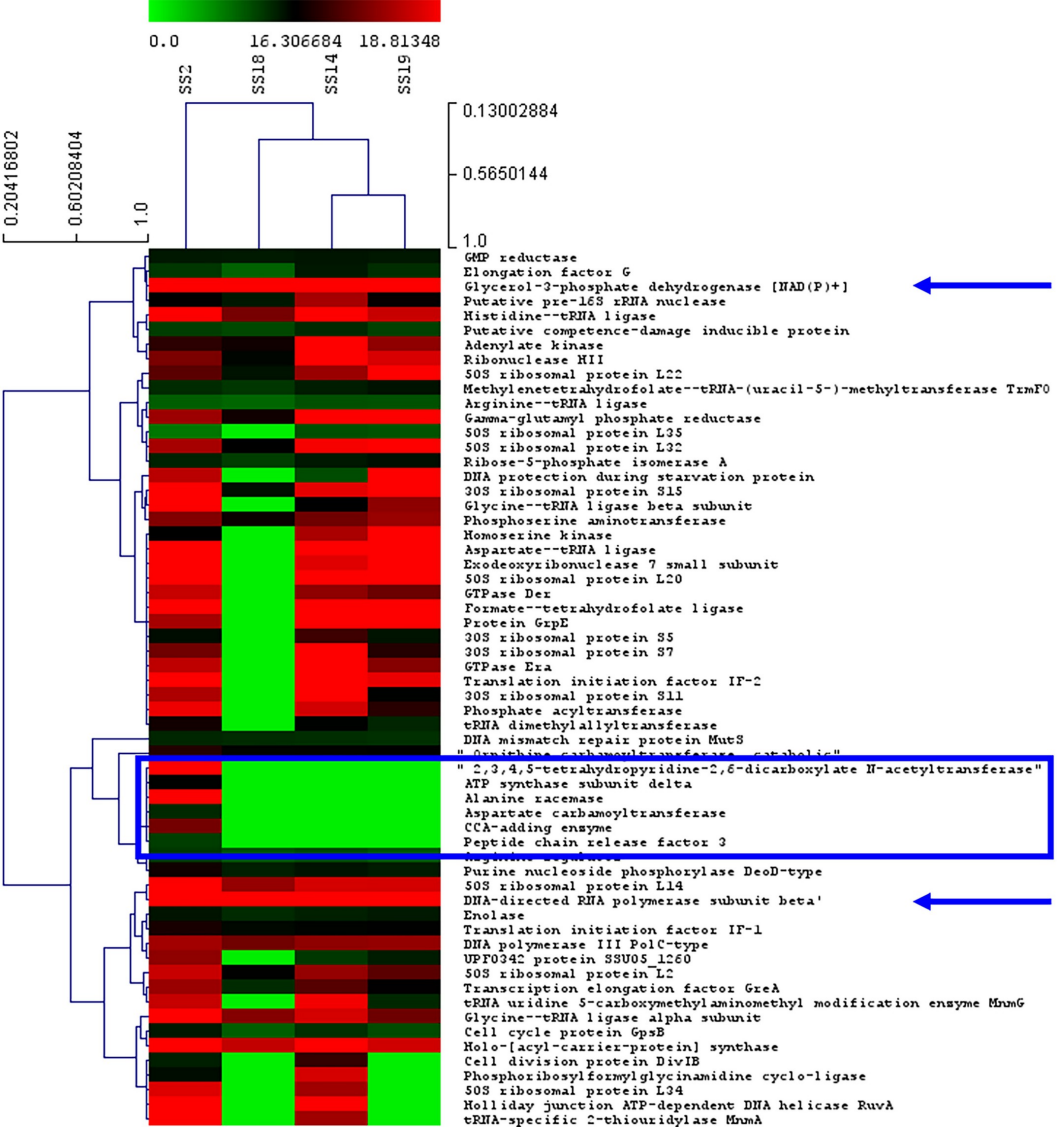
Hierarchical clustering and heat map of differentially expressed peptides in peptidomes of SS2, SS14, SS18, and SS19. Six peptides, identified only in the peptidome of SS2, in the rectangular frame were closely clustered and expressed at high to moderate levels. Two arrows specify the common peptides highly expressed across all four SS peptidomes. The green color indicates the down-regulated and the red color indicates up-regulated peptides across different serotypes.

In this study, the identified peptide expression was relatively measured using the DeCyder MS Differential Analysis Software and Mev. A gradient of peptide expression was visualized using a Hierarchical clustering heat map with 2 dimensions. The row-wise dimension is the identified proteins and the column-wise dimension is the *S*. *suis* serotypes. The green color demonstrates the down-regulated and the red color demonstrates the up-regulated peptides across four reference *S*. *suis* serotypes. In terms of the expression level across identified peptides, SS14 and SS19 were relatively more correlated than SS18 while the expression of the SS2 peptide was distantly correlated with the rest of the *S*. *suis* serotypes. SS2 and SS18 relatively expressed peptides at the highest and lowest levels, respectively. Glycerol-3-phosphate dehydrogenase [NAD(P)+] and DNA-directed RNA polymerase subunit beta were highly expressed across all four *S*. *suis* serotypes. Since six identified peptides were exclusively expressed in SS2 (Table 3), no intensity of these six identified peptides was expressed in SS14, SS18, and SS19. The top two highly expressed peptides of SS2 were 2,3,4,5-tetrahydropyridine-2,6-dicarboxylate N-acetyltransferase and alanine racemase (Fig 3). Likewise, some unidentified peptides in some *S*. *suis* serotypes demonstrated no intensity as shown in Fig 3.

## Discussion

### Peptide identification

The massess of Peptide of highly virulent SS2, less prevalent SS14, and rarely reported serotypes SS18 and SS19 extracted using the modified peptide extraction method followed by the LC-MS/MS analysis were in the range 360–3,700 Da. The peptide masses derived by the current technique provide a smaller range than the peptide mass of a previous study collected by MALDI-TOF-MS in the range of 2–20 kDa [29]. Various ranges of peptide masses mainly depend upon the different mass analyzers. The identified peptides were rather evenly distributed by either serotype (200 peptides each) or serotype grouping (2, 3, 4 serotypes in common) as shown in Fig 1. Additionally, the number of peptides as putative virulence factors was appropriate particularly to differentiate between virulent and non-virulent serotypes. Among all identified peptides, most peptides (*n* = 116) were commonly found across four *S*. *suis* serotypes. The number of co-identified proteins derived from peptidomes of virulent serotypes was five while that of serotype-specific proteins of virulent SS2 and SS14 were six and four, respectively. Therefore, the modified extraction method together with the LC-MS/MS analysis as a comparative peptidomics analysis is a suitable tool for identifying putative virulence factors of *S*. *suis*.

Most identified peptides (38.9%) from the peptidome of *S*. *suis* belong to the metabolite interconversion enzymes (Fig 2). This protein class converts one small molecule into another as opposed to the enzymes working on DNA, RNA, or a protein [34]. Approximately 33.4% of the identified peptides are translational proteins involved in the translation of mRNA to protein, while only 11.1% of the identified proteins are RNA metabolism proteins. This involves RNA processing or metabolism. The least number of identified peptides belong to the transporter protein (7%), which acts as a substance delivery system across the plasma membrane.

More than 100 peptides were commonly expressed by all four *S*. *suis* serotypes e.g. the peptide of muramidase-released protein (MRP). However, MRP was previously proposed as a virulent factor of *S*. *suis* [36]. This contradictory finding was justified by some previous studies suggesting that MRP might be unpredictably involved in *S*. *suis* pathogenesis rather than being a true virulence factor [16,37] and no correlation between the presence of virulence genes and pathogenicity could be observed [38]. Likewise, some other proteins, proposed as virulence factors of *S*. *suis* pathogenesis, were also identified in both virulent and non-virulent serotypes in the present study, such as enolase (Eno), phosphoribosylamine-glycine ligase (PurD) [16], and 30S ribosomal protein S2 (RpsB) [18]. The contradictory candidates of virulence factors proposed in previous studies could be specific to either the host or the culture condition.

SS2 and SS14 are the top two serotypes that accounted for *S*. *suis* infection in humans at the rates of 74.7 and 2.0%, respectively [6]. Therefore, peptides, commonly shared among these two virulent serotypes in the present study, are perhaps required for being virulent serotypes (Table 2). Some of these proteins are associated with the bacteria’s vital activities, such as phosphoribosyl formylglycinamidine cyclo-ligase (PurM), which is responsible for adenine and thiamine biosynthesis. The non-virulent strain *Burkholderia pseudomallei* was deficient in PurM [39]. Moreover, the Holliday junction ATP-dependent DNA helicase (RuvA) proteins, are reported to be essential for DNA helicase activity in *E*. *coli* [40]. The cell division protein (DivIB) is also responsible for bacterial cell division [41]. It is clearly shown that co-identified proteins derived from peptidomes of virulent serotypes are essentially responsible for the building blocks of bacterial DNA.

For the fact that SS2 is a highly virulent serotype in both humans and pigs, insight into the SS2-specific protein (Table 3) could shed some light on *S*. *suis* pathogenesis and its virulence factor candidates. 2,3,4,5-Tetrahydropyridine-2,6-dicarboxylate N-acetyltransferase, encoded by the *dapH* gene and ATP synthase subunit delta (F0F1-ATPases) were found in SS2 peptidome. These two enzymes involve L-lysine biosynthesis [42] and ATP synthases [43], required by bacterial cells. Despite no evidence as a virulence factor in some other bacteria, these two peptides are supposed to be candidates for the SS2 virulence factor since they are among six proteins solely identified in the SS2 peptidome (Fig 1 and Table 3).

**Fig 4:**
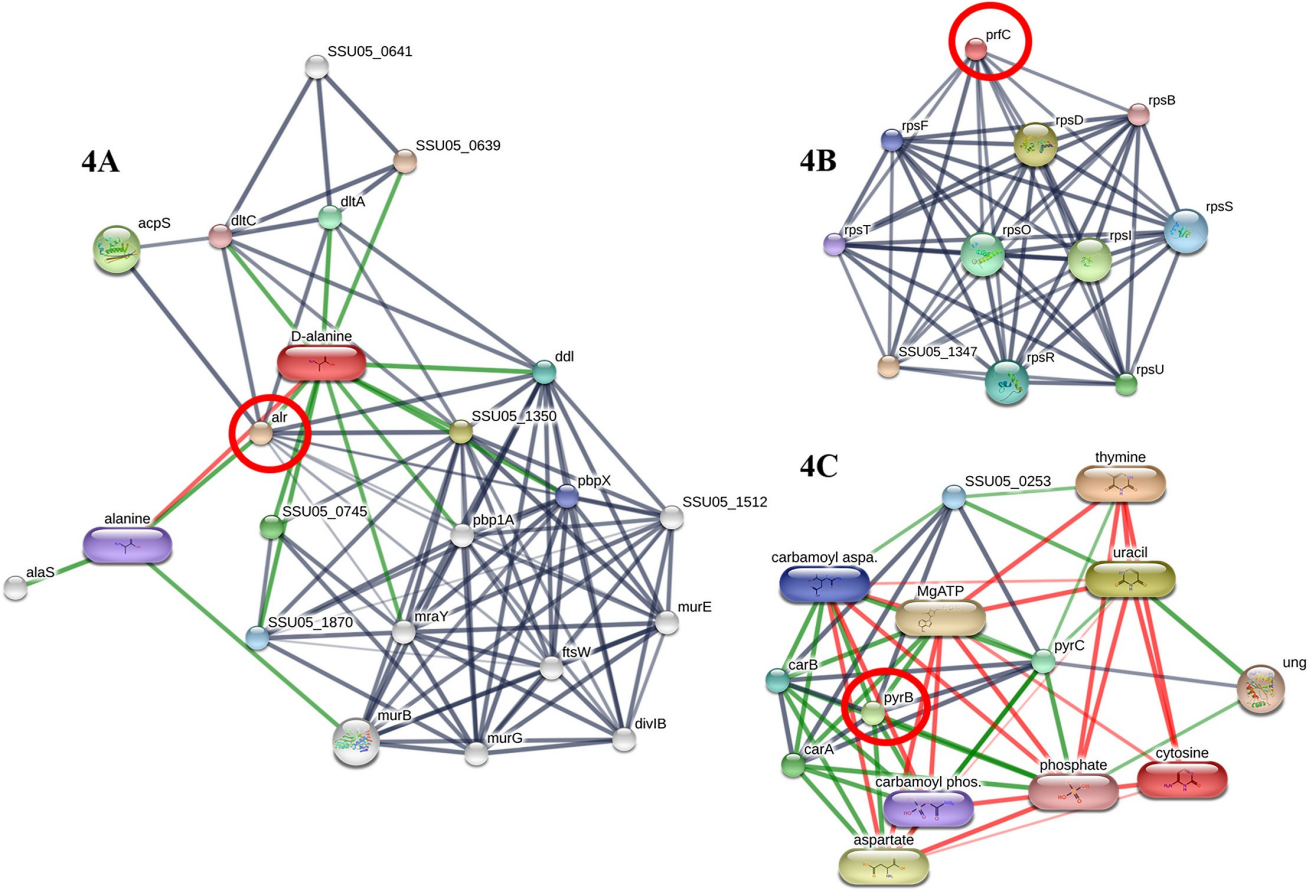
**STITCH 5.0 elucidates the association of three identified peptides derived solely from SS2 peptidome** including, Alanine racemase (Alr), responsible for the conversion of L-alanine to D-alanine, essential for peptidoglycan biosynthesis and bacterial cell wall formation (4A), Peptide chain release factor 3 (prfC) responsible for specifying the fidelity of protein synthesis, interact directly with the ribosome (4B), and ATCase (pyrB) with pyrimidine biosynthesis; nucleotides cytosine, thymine, and uracil (4C). The grey thick lines represent the highest edge confidence scores (0.900) indicating the high strength of the protein interactions at the functional level.

Identification of Alanine racemase (Alr) only in the peptidome of SS2 (Fig 1 and Table 3) suggested that SS2 possibly better replicates and persists in the host tissue than other serotypes. Since Alr is an enzyme responsible for the conversion of L-alanine to D-alanine (Fig 4A), it is essential for peptidoglycan biosynthesis followed by bacterial cell wall formation [44]. This enzyme function could enhance the cell wall integrity of *S*. *suis* to withstand the environmental harshness in the host tissue. The inhibition of Alr results in the decreased interspecies competitiveness of *Streptococcus mutants* and the pathogenicity of *Aeromonas hydrophila* [45,46]. Moreover, this enzyme has been suggested as a potential antimicrobial drug target as well [44]. Thus, we suggest that Alr is presumably significant for the virulence of SS2.

The CCA-adding enzyme or tRNA nucleotidyl transferase is a unique enzyme that plays a significant role in tRNA integrity. As tRNAs are critically important for translation, the defective tRNA must be eliminated to prevent serious consequences to the cells [47]. The CCA-adding enzyme acts by adding a degradation tag to nonfunctional tRNA so that the defective tRNA is subsequently eliminated [48]. According to our study, the CCA-adding enzyme is identified as an SS2-specific protein, considering that this highly virulent serotype exclusively encodes this enzyme to control the integrity of protein synthesis. Hence, we assume that the CCA-adding enzyme is one of the factors that strengthen the stability of SS2 cells.

Peptide chain release factor 3 or Release factor 3 (RF3) is another SS2-specific protein responsible for the integrity of protein synthesis during the elongation step of the bacterial translation [49] as shown in Fig 4B. This protein is encoded by *prfC*; the deletion of *prfC* leads to miscoding that affects both the quantity and quality of synthesized proteins in *Escherichia coli* [49]. Furthermore, this protein also plays an important role in *S*. *suis* to leverage the antimicrobial effect. The *prfC*-deleted strain of *S*. *suis* becomes more sensitive to streptomycin because of errors in the protein synthesis while wild-type *S*. *suis* is resistant to streptomycin [49–52]. In the present study, RF3 is solely identified in the SS2 peptidome (Fig 1 and Table 3). Thus, RF3 could significantly support the integrity of synthesis of SS2 proteins. With the significant primary role of RF3 in the construction of initial protein substances strengthening both structural and non-structural proteins, we assume that RF3 enables the highly virulent SS2 to survive under variable conditions including the presence of streptomycin.

Aspartate carbamoyltransferase or Aspartate transcarbamylase (ATCase), encoded by *pyrB*, is the first enzyme that plays a vital role in bacterial pyrimidine biosynthesis, resulting in nucleotides cytosine, thymine, and uracil. These are essential building blocks of DNA and RNA [53,54] as shown in Fig 4C. This enzyme is necessary for bacterial viability while the absence of *pyr*B could lead to bacterial cell death of *Helicobacter pylori* [55]. The ATCase was only identified in the peptidome of SS2 (Fig 1 and Table 3) with a moderate expression in the present culture condition. This indicates that this enzyme is relevant to SS2 virulence as SS2 is capable of modulating the level of this enzyme depending on the growth condition as ATCase was not expressed at all in the growth medium with the blood supplement.

We note that the protein expression of virulent *S*. *suis* was directly affected by the culture condition or particularly the growth medium. In our previous work using a growth medium supplemented with 5% sheep blood, the virulent-specific proteins expressed by SS2 and SS14 were the ABC-type phosphate transport system (SSU05_1106) and 30S ribosomal protein S2 (RpsB). These proteins are associated with the molecule transport system across the cell membrane and protein synthesis [18]. Whereas in the present study where *S*. *suis* was cultured in the absence of blood with the same extraction technique and peptidomics analysis, the virulent-specific proteins expressed by SS2 and SS14 were associated with genetic material and protein synthesis (Table 2). Virulent *S*. *suis* replicating under host-simulated conditions (blood supplement) dedicates its resources to responding to the host immune system (*i*.*e*. white blood cells), particularly by the first line of defense at the cell membrane. Whereas virulent *S*. *suis* cultured in the absence of blood is less defensible to the host immune system, the resource was used for the synthesis of genetic material for cell replication. No matter how different the culture condition is, the virulent *S*. *suis* is still necessary to express genes responsible for protein synthesis.

### Peptides expression

Studying the magnitude of the protein expression is an effective way to recognize which proteins are required, synthesized, and regulated by living microorganisms. Although the same peptides were identified, the level of expression can be different. Considering SS19 as a non-virulent serotype in the current study, it appeared that peptides expressed by SS14 and SS19 clusters are more correlated than those expressed by SS18 (Fig 3). SS2 is distantly clustered from the rest of the *S*. *suis* serotypes since there are five SS2-specific proteins with high to moderate levels of expression (Fig 3). The distinctive peptidomics-based phylogeny of SS2 was compatible with our previous study using MALDI-TOF-MS to differentiate 32 reference strains of *S*. *suis* [29].

Remarkably, SS2 and SS18 relatively express peptides at the highest and lowest levels, respectively. As mentioned in an earlier paragraph (Table 3), SS2 not only possesses several serotype-specific peptides supporting its existence but also expresses these proteins at a high level (Fig 3). Interestingly, the glycerol-3-phosphate dehydrogenase [NAD(P)+] and DNA-directed RNA polymerase subunit beta were highly expressed across all four *S*. *suis* serotypes. This finding wis compatible with the fact that the anaerobic condition was used to culture all *S*. *suis* serotypes and the glycerol-3-phosphate dehydrogenase [NAD(P)+] being necessary for growth in anaerobic condition [56]. The DNA-directed RNA polymerase subunit beta was involved in biofilm formation of *Enterococcus faecalis* [57] and was indicated as a virulence factor of SS2 for penetrating the blood–brain barrier in an *in vitro* co-culture model. However the serotype range in this study was limited to SS2 [58]. Considering the present study all *S*. *suis* serotypes were cultured in the absence of blood and expressed the DNA-directed RNA polymerase subunit beta at the high level. This observation might justify some primary role of this enzyme aside from it being associated with crossing the blood–brain barrier in a culture model.

Let us consider six SS2-specific peptides with a high level of expression, yet without protein expression or sharing with SS14, SS18, and SS19 at all (Fig 3 and Table 3). The top two highly expressed peptides of SS2 are Alanine racemase and 2,3,4,5-tetrahydropyridine-2,6-dicarboxylate N-acetyltransferase. These outcomes elucidate that SS2 mainly dedicated its resources to the peptidoglycan biosynthesis forming the bacterial cell wall [44] and L-lysine biosynthesis [42]. These activities are considered to be useful and potentially supportive of SS2 virulence. Similarly, the CCA-adding enzyme and peptide chain release factor 3 with a moderate level of expression are responsible for controlling the integrity of protein synthesis and then strengthening cell stability [48,49] (Fig 3). For the last two peptides, the ATP synthase subunit delta and the Aspartate carbamoyltransferase were expressed at a moderate level. These two enzymes were moderately engaged in ATP synthases [43] and bacterial pyrimidine biosynthesis [53]. Altogether, virulent SS2 prioritizes its resource to strengthen the cell wall followed by producing genetic material and energy production. This could promote its competitiveness for coexistence with some other bacteria.

A practical or advantageous application of this study is the comparison of protein expression within and between studies. The hierarchical clustering heatmap allows researchers to interpret the identified peptides or proteins more meaningfully. In the case where limited numbers of peptides were identified, the expression levels could relatively distinguish between them and explain the discrepancy. Furthermore, if possible, the comparison of protein expression could be extended across some other studies as researchers agree on a single standard of the unit of protein expression.

## Conclusions

This present study comparatively elucidated peptidomes of highly virulent SS2, less prevalent SS14, and rarely reported serotypes SS18 and SS19. The six peptides consisting of 2,3,4,5-tetrahydropyridine-2,6-dicarboxylate N-acetyltransferase (DapH), Alanine racemase (Alr), CCA-adding enzyme (CCA), Peptide chain release factor 3 (RF3), ATP synthase subunit delta (F0F1-ATPases) and Aspartate carbamoyltransferase (ATCase), identified only in peptidomes of SS2 are suggested to be associated with its virulence factor. Further study on the inhibition of these peptides should be performed to confirm their significance for *S*. *suis* virulence or pathogenesis.

## Supporting information

S1 Table The complete list of peptides derived from the peptidomes of *Streptococcus suis* serotypes 2, 14, 18, and 19. (XLSX)

## Abbreviations

ACN: Acetonitrile
Alr: Alanine racemase
ANOVA: Analysis of variance
ATCase: Aspartate carbamoyltransferase
CCA: CCA-adding enzyme
CPS: Capsular polysaccharide
Da: Dalton
DapH: 2,3,4,5-tetrahydropyridine-2,6-dicarboxylate N-acetyltransferase
DERA: Deoxyribose-phosphate aldolase
DivIB: Cell division protein
EF: Extracellular protein factor
Eno: Enolase
F0F1-ATPases: ATP synthase subunit delta
Fhb: Factor H binding protein
fMet: Methionyl-tRNA formyltransferase
HPLC: High-performance liquid chromatography
HTPA: reductase 4-hydroxy-tetrahydrodipicolinate reductase
LC-MS/MS: Liquid chromatography-mass spectrometry
MALDI-TOF MS: Matrix-assisted laser desorption/ionization time-of-flight mass spectrometry
MRP: Muramidase-released protein
MS: Mass spectrometry
NAD(P)+: Glycerol-3-phosphate dehydrogenase
PEPC: Phosphoenolpyruvate carboxylase
PurD: Phosphoribosylamine-glycine ligase
PurM: Phosphoribosyl formylglycinamidine cyclo-ligase
RbfA: Ribosome-binding factor A
RF3: Peptide chain release factor 3
RNase Y: Ribonuclease Y
RpsB: 30S ribosomal protein S2
RuvA: Holliday junction ATP-dependent DNA helicase
SLY: Suilysin
SS: Streptococcus suis serotype
SstT: Serine/threonine transporter SstT
TFA: Trifluoroacetic acid
TH kinase: Hydroxyethylthiazole kinase
tRNA: Transfer ribonucleic acid
UvrB: UvrABC system protein B
